# Mechanical Fatigue Behavior of Flexible Printed Organic Thin-Film Transistors under Applied Strain

**DOI:** 10.3390/ma10010018

**Published:** 2016-12-28

**Authors:** Tomohito Sekine, Daisuke Kumaki, Shizuo Tokito

**Affiliations:** Research Center for Organic Electronics (ROEL), Graduate School of Science and Engineering, Yamagata University, 4-3-16 Jonan, Yonezawa, Yamagata 992-8510, Japan; d_kumaki@yz.yamagata-u.ac.jp (D.K.); tokito@yz.yamagata-u.ac.jp (S.T.)

**Keywords:** organic thin-film transistor, mechanical fatigue, flexible, printed, strain

## Abstract

We report on the mechanical fatigue behavior of printed, organic, thin-film transistors (OTFTs) based on a polymer semiconductor, investigated by repeatedly applying strain to the flexible OTFT devices and assessing their electrical characteristics after 60,000 bending cycles. As part of our investigation, we established that the rates of reduction in source/drain currents in the OTFT device depended on bending directions. Our improved understanding of the mechanical fatigue behavior of the flexible printed OTFT devices provides valuable insights into their employment in practical flexible electronics applications.

## 1. Introduction

Flexible printed organic thin-film transistor (OTFT) devices have been attracting significant attention in the development of emerging flexible electronic applications, as they possess multiple advantages compared to the conventional silicon TFT devices in several applications, such as organic electroluminescence devices [1,2,3], logic circuits [4], and sensors [5,6]. Moreover, fabrication processes based on printing technology can dramatically improve material utilization and reduce waste, while minimizing processing costs compared to the conventional photolithography and vacuum deposition processes; they can also be readily scaled to large areas [7,8,9].

Providing for mechanically robust, flexible, printed OTFT devices is an important issue in the development of flexible, printed, organic electronics. Previously reported results by several research groups confirmed that electrical characteristics of the OTFT devices are sensitive to applied mechanical stress [10,11,12,13]. However, few discussions of the mechanical fatigue behavior on the OTFT devices are established. Moreover, most of these studies used evaporated metals and organic semiconducting materials and did not utilize printing processes [14,15,16,17]. In particular, no study has been carried out on the relationship between the mechanical fatigue and changes in electrical characteristics of the OTFT devices. In fact, although we confirmed that the OTFT devices, based on a polymeric semiconductor, are more stable under mechanical stress than a small-molecule OTFT device [18,19], few attempts have been made to understand their behavior under mechanical fatigue. This improved understanding of the mechanical fatigue behavior of the OTFT device is useful for their employment in practical flexible electronics applications.

In this report, the mechanical fatigue behavior on flexible printed OTFT devices was investigated by repeatedly applying strain. We established that reduction rates of a source/drain current (*I*_DS_) in OTFT devices depends on bending directions and the number of bending cycles. Moreover, it was revealed that reductions in *I*_DS_ were caused at the interface between source/drain electrodes and a semiconducting layer of the OTFT devices.

## 2. Results and Discussion

**Figure 1:**
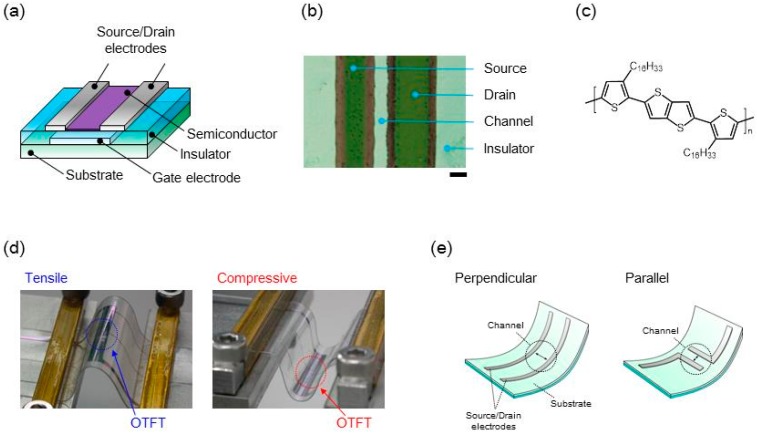
Flexible printed organic thin-film transistor (OTFT) device. (**a**) Schematic illustration of the OTFT device; (**b**) Optical microscope image of the fabricated OTFT device in the channel region. Scale bar, 100 µm; (**c**) Chemical structure of semiconducting material used in this study; (**d**) Photographs illustrating the method of strain application to the OTFT devices, (left) tensile strain, (right) compressive strain; (**e**) Bending direction of the flexible OTFT device.

Figure 1a shows a schematic illustration of bottom-gate and bottom-contact OTFT devices that we fabricated. In order to improve mechanical durability of the printed OTFT device, we used poly[2,5-bis(3-hexadecylthiophen-2-yl)thieno[3,2-b]thiophene] (PBTTT-C16) as the organic semiconducting layer (Figure 1c) [20,21]. The procedure used for applying strain to the OTFT devices is explained in the experimental section of this paper.

**Figure 2:**
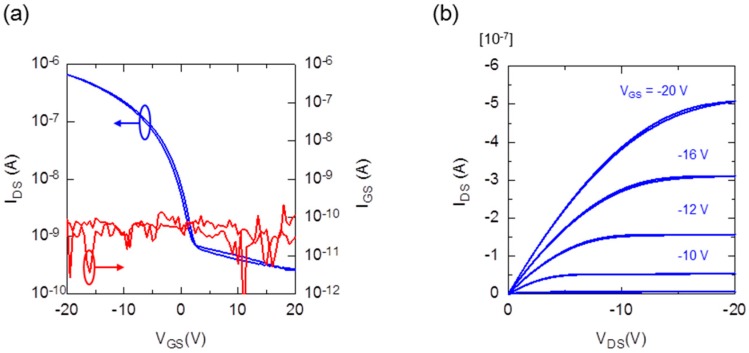
(**a**) Transfer characteristics of the fabricated OTFT device. The OTFT devices were monitored at *V*_DS_ = −20 V, *V*_GS_ = +20 to −20 V, and W/L = 1000/60 µm; (**b**) Corresponding output characteristics. The plot is *I*_DS_ as a function of *V*_DS_ for *V*_GS_ from −10 V to −8 V in 2 V steps.

Figure 2 shows typical transfer and output characteristics for the flexible printed OTFT devices based on PBTTT-C16. The *I*_DS_ was measured as a function of the gate/source voltage (*V*_GS_) at a constant source/drain voltage (*V*_DS_) of −20 V in a nitrogen atmosphere (Figure 2a). The estimated field-effect mobility (μ_eff_) in the saturation region for the OTFT devices was 0.03 cm^2^·V^−1^·s^−1^ and the on/off current ratio exceeded 10^3^. The output curve shows a distinct saturation region when high gate voltages (*V*_GS_) were applied to the OTFT devices (Figure 2b). As the result, the PBTTT-C16-based OTFT devices with printed electrodes exhibited reasonable electrical performance on a flexible substrate.

**Figure 3:**
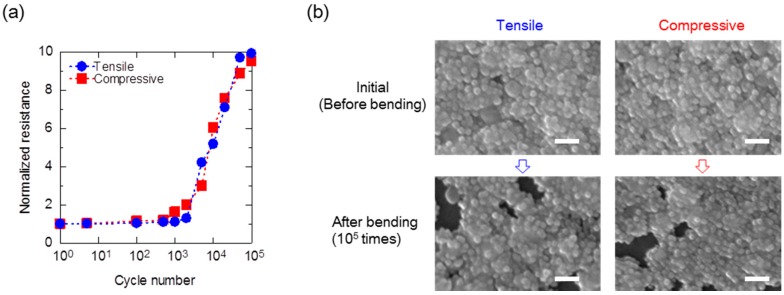
(**a**) Normalized resistivity of a printed electrode as a function of the number of bending cycles. The obtained data were normalized to resistivity value before bending; (**b**) Surface SEM images of the printed electrode before and after strain application. Scale bar, 100 nm.

Stress cycling tests were carried out on a printed electrode and PBTTT-C16-based OTFT devices, whereby the devices were bent from their flat state to 1.5% tensile or compressive strain and then released to their flat state again. Figure 3a shows the normalized resistivity of the printed silver source/drain electrodes as a function of the number of bending cycles. The lines in the plots serve as visual guides. The initial resistivity of the printed electrode was 7 μΩ·cm, which is the same order as bulk silver [22,23]. After the stress cycling 10^5^ times, the resistivity increased only ten-fold, to approximately 70 μΩ·cm. Although the resistivity of the printed electrodes deteriorated with the applied strain, the rate of change was small. In fact, we checked a surface morphology of the printed electrode before and after the stress cycling testing by using a scanning electron microscope (SEM) (JEOL JSM7600-FE, Tokyo, Japan) (Figure 3b). There were few cracks in the surface of the printed electrodes after tensile and compressive strain applied for 10^5^ cycles. Thus, it appeared that the changes of resistivity of the printed electrodes did not cause changes in the OTFT devices’ characteristics.

**Figure 4:**
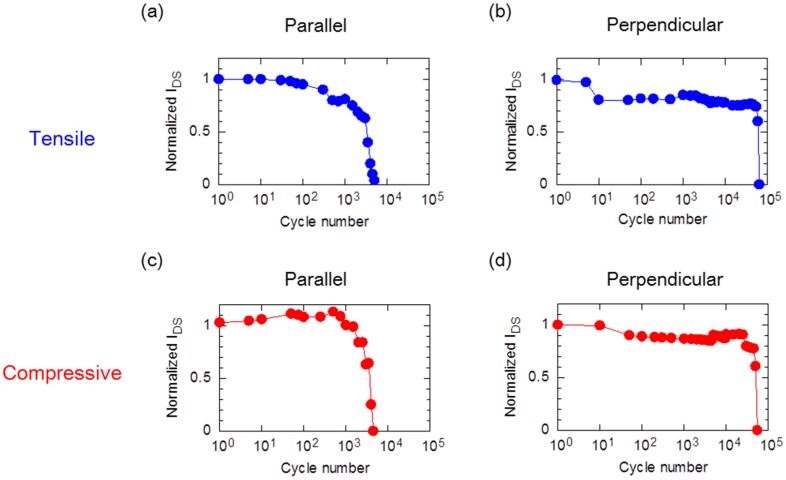
Electrical characteristics of the OTFT devices under mechanical fatigue (**a**) tensile strain with parallel and (**b**) perpendicular bending directions; and (**c**) compressive strain with parallel and (**d**) perpendicular bending directions.

Stress cycling tests were also carried out on flexible printed OTFT devices. Figure 4 shows the changes of the *I*_DS_ of the PBTTT-C16-based OTFT devices after 1.5% tensile and compressive strain repeatedly applied. The OTFT devices were monitored at *V*_DS_ = −20 V, *V*_GS_ = +20 to −20 V. The *I*_DS_ was normalized to the initial value of *I*_DS_ with the application of no strain at *V*_GS_ = −10 V. The lines in the plots serve as visual guides. After applying a tensile strain, the *I*_DS_ of the OTFT devices showed decreases that depended on the number of bending cycles in parallel or perpendicular directions to the current flow (Figure 4a,b). Specifically, in Figure 4, the strain shifted Vth of the OTFT devices, which occurred by applying the strain to the PBTTT-C16 OTFT devices. In this paper, we excluded the value of Vth shifts by calculating the electric characteristics of OTFT devices. Bending in the perpendicular direction demonstrated higher mechanical durability than in the parallel direction, which underwent 60,000 bending cycles. After the application of compressive strain to the OTFT devices, the behavior of the *I*_DS_ decreases showed the same tendency as for tensile strain (Figure 4c,d). Incidentally, the *I*_GS_ values of the OTFTs before applied strain are the following; tensile strain of parallel: 2 × 10^−10^ A, perpendicular: 3 × 10^−10^ A, compressive strain of parallel: 1 × 10^−10^ A, perpendicular: 8 × 10^−10^ A. The values of *I*_GS_ did not surpass 1 × 10^−10^ A after applied strain. As a result, we found that the mechanical fatigue behavior of the OTFT devices depended on only the bending direction: parallel or perpendicular.

**Figure 5:**
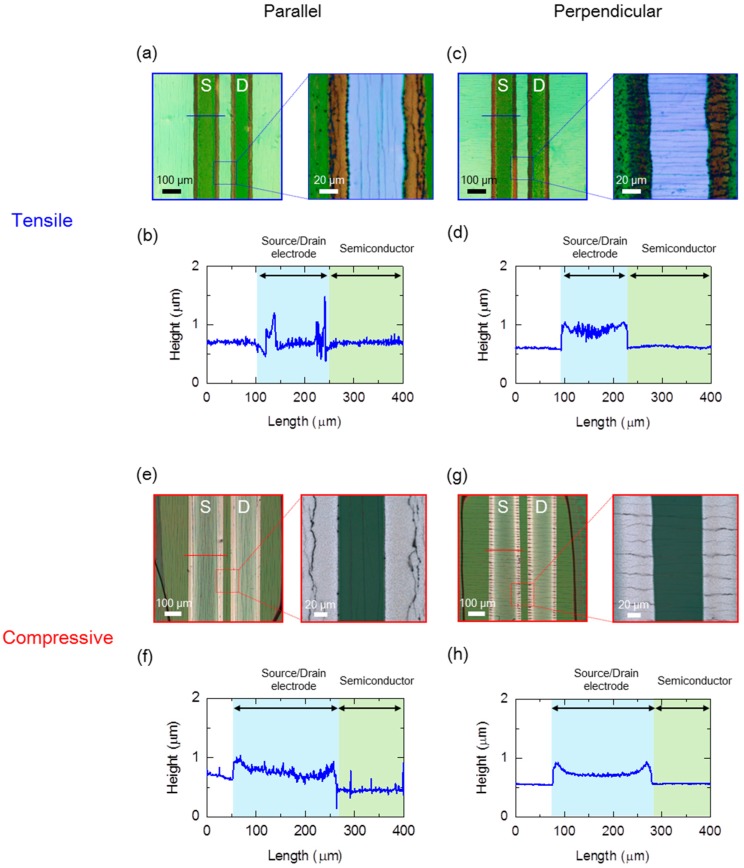
Strain-induced surfaces and cross-sections of the OTFT devices. (**a**–**d**) Top-view photos (top), and sectional profile (bottom) of the OTFT device near the channel region after application of tensile strain, (**a**,**b**) parallel bending, (**c**,**d**) perpendicular bending; (**e**–**h**) Top-view photos (top), and sectional profile (bottom) of the OTFT device near the channel region after applying compressive strain, (**e**,**f**) parallel bending, (**g**,**h**) perpendicular bending. (**a**,**c**,**e**,**g**) Inset bars are the observed points in the sectional profiles.

To understand the cause of the decreases in the *I*_DS_ for the OTFT devices, their surface morphology was investigated using a laser microscope (Olympus OLS-4000, Tokyo, Japan) before and after stress cycles testing. Figure 5 shows the surface topography and cross-sectional profiles of the OTFT devices near the channel region after applying tensile and compressive stains for each bending direction. We showed the optical image of the OTFT devices before applied strain in Figure 1b. When applying tensile strain, cracks appeared along an edge of the source/drain electrode when the bending was applied to the device in the parallel direction (Figure 5a). In fact, these cracks interrupted the electrode and the semiconducting layer after applying the tensile stress (Figure 5b). On the other hand, when bending in the perpendicular direction, cracks appeared almost uniformly across the channel (Figure 5c). Unlike for the parallel bending, these cracks did not appear at the interface between the source/drain electrodes and the semiconducting layer (Figure 5d). The fact that topography was preserved at the interface could be the cause of saved *I*_DS_ in the OTFT devices after applying strain. Likewise, changes in the topology of the OTFT devices after applying compressive strain in each bending direction exhibited the same tendency as the results when applying tensile strain (Figure 5e–h). These changes in topography corresponded to the reductions in the *I*_DS_ for the OTFT devices. These results indicate that there is a correlation between reduced *I*_DS_ and damage to the OTFT device structure. In particular, we learned that bending direction affected the amount of the *I*_DS_ reduction by the mechanical fatigue of the OTFT devices. This study found that accumulation of mechanical stress at the interface between the source/drain electrodes and the semiconducting layer of the OTFT devices is the cause of damage in the flexible printed OTFT devices. In addition, selection of an optimal material for the source/drain electrodes improves the mechanical fatigue behavior in OTFT devices. In fact, Fukuda et al. recently discussed how the optimization of electrode materials can improve the mechanical durability when applying strain [24]. We will explore the use of better optimized OTFT device electrode materials in a follow-up study.

## 3. Experimental Section

### 3.1. Device Fabrication

The OTFT devices used in this study were fabricated on poly(ethylene naphthalate) (PEN) film substrates (Teijin DuPont Films Teonex^®^, 125 µm, Tokyo, Japan). A cross-linkable poly(4-vinylphenol) (PVP) (Sigma-Aldrich 436224, Tokyo, Japan) solution consisting of a mixture of PVP and melamine resin (Sigma-Aldrich 418560), using 1-methoxy-2-propyl acetate (PEGMEA) (Kanto Chemicals 01948-00, Tokyo, Japan) as a solvent, was spin-coated onto the PEN film as a planarization layer. After a 30 nm-thick Al gate electrode was deposited onto the planarization layer, a 300 nm layer of polyparaxylene [25,26] was deposited as the gate insulator. Source and drain electrodes were then formed with a silver nanoparticle ink (Harima Chemicals NPS-JL, Tokyo, Japan) using an inkjet printer (Fujifilm Dimatix DMP-2831) onto the gate insulator, and sintered at 150 °C for 1 h. Fluoropolymer (DuPont Teflon AF1600) bank layers (200 nm thick) were printed using dispenser equipment (MUSASHI Engineering Image Master 350 PC, Tokyo, Japan) at a patterning speed of 20 mm·s^−1^ and with a discharge pressure of 5 kPa. During the dispenser patterning process, the platen and nozzle temperatures were kept at 30 °C. After printing the bank layers, the substrates were stored in an air ambient for 10 min to remove the solvent. Finally, a semiconducting polymer PBTTT-C16 (Merck) (0.03 wt %) in toluene (Kanto Chemicals 40500-05) was drop-coated onto the insulator layer as the semiconducting layer and annealed at 150 °C for 1 h in a dry nitrogen atmosphere. The nominal channel length and channel width for the OTFT device were 60 µm and 1000 µm respectively. The electric characteristics were measured in dry nitrogen using a semiconductor parameter analyzer (Keithley, model 4200-SCS).

### 3.2. Calculating Strain in the OTFT Device

In this study, tensile and compressive strain were applied to the printed flexible OTFT devices in directions perpendicular and parallel to the direction of the current flow between the source/drain electrodes (Figure 1d,e), and the transistor characteristics were evaluated before and after the application of strain. The surface strain induced on the active layer was estimated using the following formula [18,27,28]:

$$S=\frac{d_{s}}{2R}$$

where *d_s_* and *R* are the thickness of the substrate and the bending radius, respectively. We applied the tensile and compressive strain with bending radii as small as 4 mm, corresponding to an induced surface strain of approximately 1.5%. The OTFT device performance under mechanical fatigue was evaluated after removing strain from the device.

## 4. Conclusions

We have reported on the mechanical fatigue behavior of flexible printed OTFT devices with PBTTT-C16 semiconducting materials on plastic film substrates. Changes in OTFT device electrical characteristics after applying strain depended on the number of bending cycles and the direction of the strain, perpendicular or parallel to the current flow. These insights are useful in the practical applications of OTFT devices to flexible electronics applications.
